# The Beacon Light

**Published:** 1924-03

**Authors:** 


### THE "BEACON LIGHT."

The St. Marylebone Hospitals League, the foundation of which was cordially welcomed in these columns, continues to grow, and the latest sign of its activity is a monthly journal, called the "Beacon Light," the first two issues of which lie before us. The League differs from similar bodies in several interesting ways. It represents the eight hospitals of the borough, and seeks to enrol regular subscribers from the firms and shops and offices that crowd this central district of London. But it is much more than a collecting agency. It is also a social and educational one, whose members are brought into personal contact with the work of the hospitals, and can attend many lectures and demonstrations in the institutions themselves. This is a highly important and much appreciated departure, which gives to the members of the League some of the privileges of a society or college and redeems the whole scheme from mere collecting. Not only are these demonstrations and scientific evenings regularly held, but First Aid instruction is given, through the League, to chosen representatives of the firms whose work involves the risk of accidents, and those who have been instructed will work in association with the casualty officers at the different hospitals. Besides this the board rooms at the different hospitals are nightly at the disposal of the various committees, and all the organisers of the scheme, which is divided into eight areas within the borough, are welcomed to them.

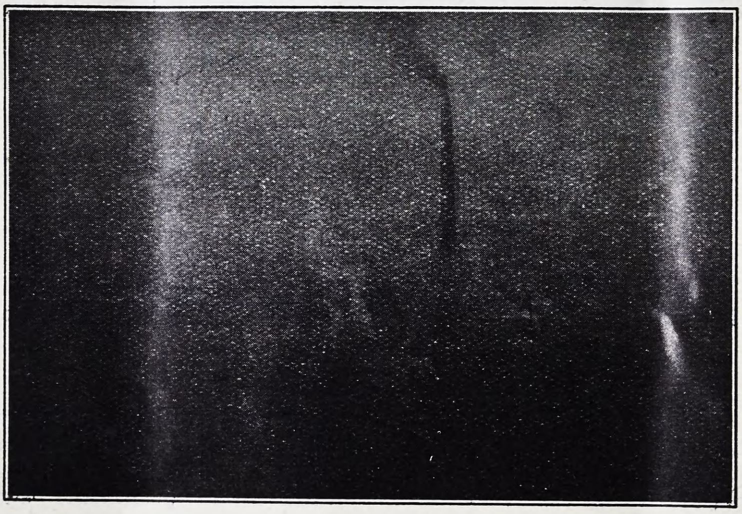

Sheffield: 4.45 p.m., Monday.

### Rapid Progress.

Already 250 firms have joined the League with a total of 7,000 members, and the number of members is said to be growing at the rate of several hundreds a week. It is also hoped that each Area Committee will arrange its own social evenings to encourage the good fellowship of the members and possibly to provide for the administration expenses incurred. We believe that the educational and social side, with the hospitals for its centre, is very useful, and may come to be regarded as a privilege open to all members, in their days of health, just as treatment in hospital is after an accident or during illness. Those who attend the demonstrations will be interested and informed concerning not only the work of the hospitals but the nature of disease and the points on which research is being undertaken. All this cannot fail to benefit themselves, and they will incidentally learn from it what is the work that they are supporting and the part the hospitals play in civic life. Such knowledge, once imparted, does not fade, and is probably the only permanent basis for public support of the voluntary system.

### Hospitals as Educational Centres.

There should, then, be abundant material for the "Beacon Light" to draw upon, and since it will come into the hands of many doubtful or prospective members, each number should include some account of the most interesting lecture or demonstration given during the previous month, together with the programme of forthcoming evenings. The paper should be as interesting as the lectures. The League embodies what is virtually a new principle in hospital support, and if it proves successful will set a valuable example that may lead to every hospital becoming an educational centre to its supporters.

### PRACTICE IN SOUTH AFRICA.

We notice that, according to the *South African Medical Record*, medicine in the Union is no more exempt than the corresponding sciences in this country from the public effects of that style of journalism which aims at tickling the jaded palate of the reader and remains quite careless whether the statements it makes are even remotely in accordance with the facts. According to certain non-medical articles appearing in the South African Press one might easily be led to believe that there was something like an appalling scarcity of medical practitioners in that admirable country. This, however, is by no means the case. There has, in fact, been in recent years a noticeable tendency for non-South African practitioners to return home to this country. The difficulties of the visitor need no description; not the least of these is the necessity to be bi-lingual

